# B-Boxing beats the heat for lilies

**DOI:** 10.1093/plphys/kiag178

**Published:** 2026-04-03

**Authors:** Maneesh Lingwan

**Affiliations:** Assistant Features Editor, Plant Physiology, American Society of Plant Biologists, Rockville, MD 20855-2768, United States; Donald Danforth Plant Science Center, St.Louis, MO 63132, USA

In the era of global climate instability, heat stress (HS) has become a major abiotic factor that undermines plant productivity and survival. Ornamental crops such as *Lilium longiflorum* (lily) are key species in the global floriculture industry and are vulnerable to high temperatures, which impose physiological burdens, reduce biomass, and compromise floral quality (Zhou et al. 2022). Heat shock transcription factors (HSFs) act as master regulators under stress and activate the expression of Heat Shock Proteins (HSPs), which are recognized as the main components of the thermotolerance pathway (Bakery et al. 2024) (Fig. 1). Recent genomic and proteomic studies have emphasized the importance of the B-box (BBX) family proteins, a group of zinc-finger transcription factors known for their roles in light signaling and HS responses (Yadav et al. 2019; Li et al. 2025). However, the regulatory networks that can improve HS tolerance remain poorly understood.

**Figure 1:**
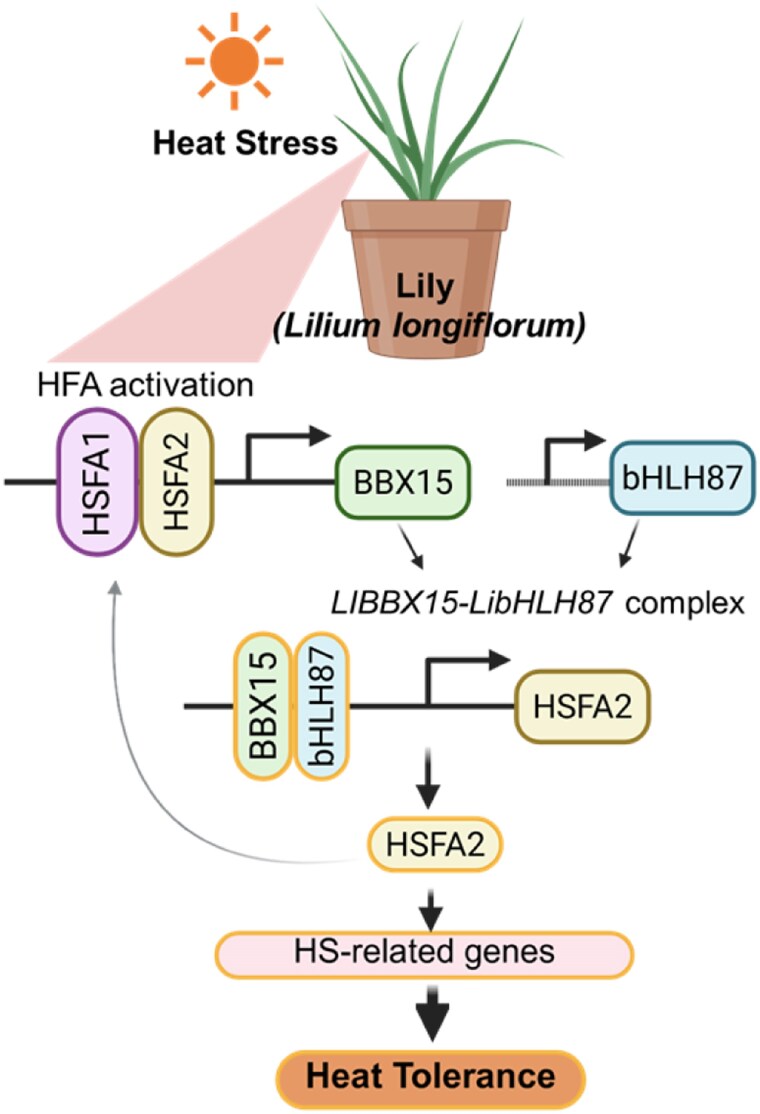
Illustration of *LlBBX15*-mediated heat stress responses in lily. HS activates the master regulators LlHSFA1 and LlHSFA2 transcription factors, which directly bind to the *LlBBX15* promoter to quickly induce its transcription. LlBBX15 forms a functional complex with the transcription factor LlbHLH87. This complex targets the *LlHSFA2* promoter, significantly increasing its expression and creating a strong feed-forward loop. Elevated *LlHSFA2* levels create feedback loops to maintain high *LlBBX15* expression while also promoting the transcription of downstream HSPs and HS tolerance. The figure is adapted from Wu et al. (2026) and visualized using BioRender.

In the current issue of *Plant Physiology*, Wu et al. (2026) identify LlBBX15 as a key heat-inducible BBX protein in lily. LIBBX15 interacts with basic helix-loop-helix (bHLH) proteins, a superfamily of transcription factors that bind as dimers to specific DNA target sites to coordinate the thermotolerance network. BBX15 is a Class III B-box subfamily protein with a conserved N-terminal B-box domain and a C-terminal CCT domain. *LlBBX15* mRNA is strongly induced within 30 min of thermal stress, and the mRNA remains elevated for up to 12 h. Subcellular studies using 35S promoter-driven GFP-tagged proteins in *Nicotiana benthamiana* revealed that *GFP-LlBBX15* is localized in the nucleus, regardless of temperature. Transactivation assays in yeast and dual-luciferase assays in *N. benthamiana* confirmed that *LlBBX15* is a potent transcriptional activator, and its transactivation activity is confined to a specific region of the B-box and CCT domains.

HSFA2 is known as a central player in thermotolerance acquisition in Arabidopsis (Ohama et al. 2017); however, its direct regulator was not known. The yeast one-hybrid assay demonstrates that *LlBBX15* directly binds to the *LlHSFA2* promoter. Electrophoretic mobility shift assays further demonstrates that *LlBBX15* binds strongly to the *LlHSFA2* promoter. BBX proteins are known to interact with bHLH transcription factors (Cao et al. 2023). In a previous study, the authors observed that *LlbHLH87* expression is induced by HS and acts as an activator of *LlHSFA2* (Wu et al. 2024), suggesting that LlBBX15 might also interact with LlbHLH87. Further, integrating yeast 2-hybrid screening, bimolecular fluorescence complementation and luciferase complementation imaging assays confirmed a direct physical interaction between LlBBX15 and LlbHLH87. Both proteins can form homomeric interactions through their conserved motifs, but the formation of a heterocomplex LlBBX15-LlbHLH87 significantly stabilizes their respective homomeric interactions. This mutual stabilization enhances the overall stability of the transcriptional complex. The dual-luciferase reporter assay showed that both LlBBX15 and LlbHLH87 independently activated the *LlHSFA2* promoter. Co-overexpression of *LlBBX15* and *LlbHLH87* resulted in significantly greater thermotolerance than overexpression of BBX15 alone, suggesting that LlBBX15 coordinates with LlbHLH87 to form a high-affinity regulatory module to improve thermotolerance.

Wu et al. (2026) further investigated the physiological role of BBX15, demonstrating that overexpression of *LlBBX15* increased heat survival rates in both Arabidopsis and Lily plants. Under heat stress, *LlBBX15* overexpression plants showed reduced ion leakage, improved membrane integrity, and retained higher chlorophyll content than wild-type plants. Further, TRV-mediated virus-induced gene silencing of *LlBBX15* led to lily plants being more heat sensitive and accelerated leaf yellowing, wilting, and cellular damage. The author also showed that heat stress transcription factors LlHSFA1 *and* LlHSFA2 directly regulated *LlBBX15* and synergistically activated its expression. In summary, LlBBX15 serves as a molecular link between the bHLH family and the HSF signaling pathway, coordinating a positive role in thermotolerance.

## Relevant articles in *Plant Physiology*:

- Mukherjee et al. (2026) compiled evidence of BBX-mediated light regulation of the seed-to-seedling transition under dynamic environmental conditions.
- Chen et al. (2022) reported that BBX7 confers drought tolerance in apple.
- Job et al. (2022) showed that the transcription factor BBX11 regulates UV-B tolerance in Arabidopsis.

## Data Availability

No new data were generated or analyzed in support of this research.
